# Mining *Aegilops tauschii* genetic diversity in the background of bread wheat revealed a novel QTL for seed dormancy

**DOI:** 10.3389/fpls.2023.1270925

**Published:** 2023-11-30

**Authors:** Monir Idres Yahya Ahmed, Yasir Serag Alnor Gorafi, Nasrein Mohamed Kamal, Mohammed Yousif Balla, Izzat Sidahmed Ali Tahir, Lipeng Zheng, Naoto Kawakami, Hisashi Tsujimoto

**Affiliations:** ^1^ United Graduate School of Agricultural Sciences, Tottori University, Tottori, Japan; ^2^ International Platform for Dryland Research and Education, Tottori University, Tottori, Japan; ^3^ Gezira Research Station, Agricultural Research Corporation (ARC), Wad-Medani, Sudan; ^4^ Arid Land Research Center, Tottori University, Tottori, Japan; ^5^ Department of Life Sciences, School of Agriculture, Meiji University, Kawasaki, Japan

**Keywords:** bread wheat, *Aegilops tauschii*, gene mining, seed dormancy, QTL analysis, germplasm enhancement

## Abstract

Due to the low genetic diversity in the current wheat germplasm, gene mining from wild relatives is essential to develop new wheat cultivars that are more resilient to the changing climate. *Aegilops tauschii*, the D-genome donor of bread wheat, is a great gene source for wheat breeding; however, identifying suitable genes from *Ae. tauschii* is challenging due to the different morphology and the wide intra-specific variation within the species. In this study, we developed a platform for the systematic evaluation of *Ae. tauschii* traits in the background of the hexaploid wheat cultivar ‘Norin 61’ and thus for the identification of QTLs and genes. To validate our platform, we analyzed the seed dormancy trait that confers resistance to preharvest sprouting. We used a multiple synthetic derivative (MSD) population containing a genetic diversity of 43 *Ae. tauschii* accessions representing the full range of the species. Our results showed that only nine accessions in the population provided seed dormancy, and KU-2039 from Afghanistan had the highest level of seed dormancy. Therefore, 166 backcross inbred lines (BILs) were developed by crossing the synthetic wheat derived from KU-2039 with ‘Norin 61’ as the recurrent parent. The QTL mapping revealed one novel QTL, *Qsd.alrc.5D*, associated with dormancy explaining 41.7% of the phenotypic variation and other five unstable QTLs, two of which have already been reported. The *Qsd.alrc.5D*, identified for the first time within the natural variation of wheat, would be a valuable contribution to breeding after appropriate validation. The proposed platform that used the MSD population derived from the diverse *Ae. tauschii* gene pool and recombinant inbred lines proved to be a valuable platform for mining new and important QTLs or alleles, such as the novel seed dormancy QTL identified here. Likewise, such a platform harboring genetic diversity from wheat wild relatives could be a useful source for mining agronomically important traits, especially in the era of climate change and the narrow genetic diversity within the current wheat germplasm.

## Introduction

Agricultural production worldwide is expected to be adversely affected by climate change. Temperatures are expected to rise, and the frequency of heat waves has steadily increased in recent years. Moreover, rainfall is becoming unpredictable with many drought or heavy rainfall events and erratic distribution (Elahi et al., 2022). Under this complicated situation, it is imperative to develop new crop varieties that can withstand these erratic weather conditions. However, this is very challenging in wheat due to the narrow genetic diversity associated with decades of extensive breeding. Therefore, in order to develop new cultivars in the era of climate change, it is important to identify new sources of novel genes or alleles for wheat breeding.

*Aegilops tauschii* (2n = 14, DD) is considered a valuable source of novel alleles for improving bread (common) wheat (*Triticum aestivum*, 2n = 42, AABBDD) cultivars (Singh et al., 2019). Currently, the common wheat germplasm does not adequately represent the genetic diversity of *Ae. tauschii* because common wheat originated from interspecific hybridization events between tetraploid wheat and only a limited number of *Ae. tauschii* plants in a certain distribution area. Breeders have used several methods to enrich the genetic diversity of common wheat using *Ae. tauschii*. The most common route involves hybridization between tetraploid wheat and *Ae. tauschii* to create primary synthetic hexaploids (Li et al., 2018). Several superior genes from *Ae. tauschii* have been transferred into common wheat through synthetic wheat (Dale et al., 2017).

Nevertheless, evaluating yield potential traits at the synthetic wheat level is challenging because of the plant shape and spike morphology, and the expected traits may not always appear in progenies because of considerable genetic differences between synthetic wheat and elite wheat cultivars (Ogbonnaya et al., 2013). To overcome these challenges, diverse *Ae. tauschii* genes should be “diluted” and then evaluated in the genetic background of elite wheat cultivars (Ogbonnaya et al., 2013). Therefore, Tsujimoto et al. (2015) proposed a population of multiple synthetic derivatives (MSD) as a new tool to evaluate the *Ae. tauschii* genes in the background of hexaploid wheat. This mixed population was developed by crossing and backcrossing the wheat cultivar (Norin 61) and several primary synthetic hexaploid lines (Matsuoka and Nasuda, 2004). Gorafi et al. (2018) with selected 400 lines demonstrated the possibility of identifying the pedigree of the lines in this mixture population using DArTseq molecular markers and confirmed the suitability of the population for genetic studies. However, no systematic study used this highly diverse population to identify specific trait phenotypes and their underlying QTLs or genes from larger number of individuals.

Pre-harvest sprouting (PHS) it known to decrease grain yield and end-use quality due to the breakdown of starch and proteins, resulting in severe annual wheat yield losses of about one billion US dollars worldwide (Shorinola et al., 2016; Shao et al., 2018). Among many factors that linked to PHS resistance, seed dormancy characteristic is the most critical one (Dale et al., 2017). As a complex trait, seed dormancy is affected by genetic factors, environmental conditions and their interaction (Jaiswal et al., 2012; Kulwal et al., 2012). Because high selection pressures were imposed in wheat breeding programs against seed dormancy to achieve uniform and rapid seed germination, most of the modern commercial wheat cultivars are disposed to preharvest sprouting (Meyer and Purugganan, 2013; Gao and Ayele, 2014). Recently, due to unpredictable weather conditions associated with climate change, wheat breeding programs in many areas around the world have developed interest in breeding wheat cultivars with a higher level of seed dormancy. Revisiting the wheat wild relatives might be one of the best strategies to restore the seed dormancy in wheat. For instance, major QTLs for seed dormancy from *Ae. tauschii* were identified through advanced backcross population developed by means of synthetic octaploid wheat (Dale et al., 2017). However, so far, limited number of *Ae. tauschii* have been used. Therefore, using a wide range of *Ae. tauschii* may be a promising method to restore dormancy in the modern wheat cultivars.

In this study, we explain how we successfully identified a novel QTL for seed dormancy through systematic evaluation of the *Ae. tauschii* genes in the background of hexaploid wheat using the mixed population of the MSD lines and developed backcross inbred lines population. This study demonstrates a practical example of efficient mining of *Ae. tauschii* genetic diversity using the MSD platform, which can be used to uncover other novel QTLs and genes associated with abiotic stress tolerance for developing climate-resilient wheat cultivars.

## Materials and methods

### Population of MSD lines

**Figure 1:**
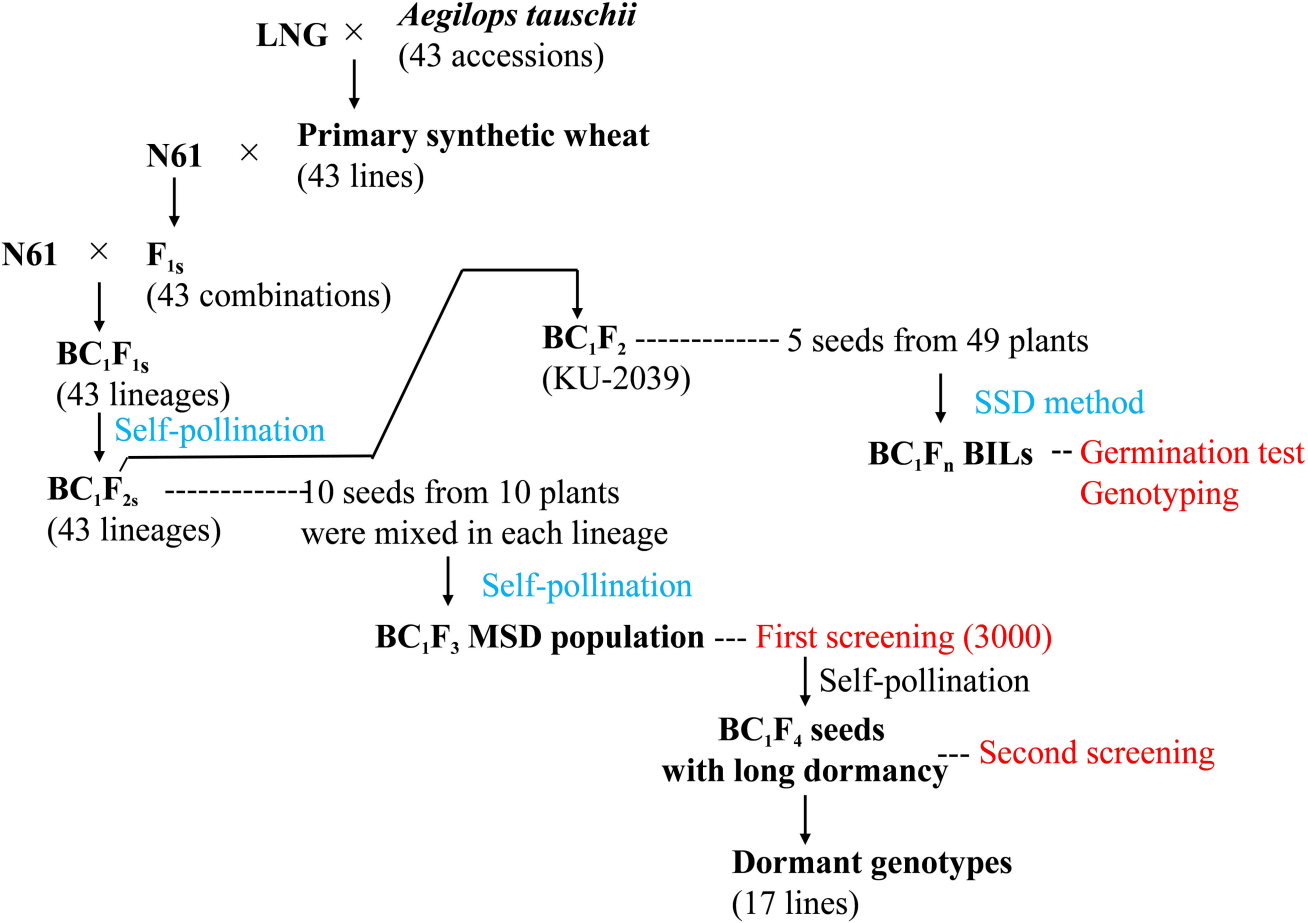
Pedigrees of plant materials and steps to identify dormant genotypes in the population of multiple synthetic derivatives (MSD) and backcross inbred lines (BILs). LNG: *Triticum durum* cv. ‘Langdon’; N61: *T. aestivum* cv. ‘Norin 61’; SSD, single seed descent.

To identify lines with dormant seeds, we used an MSD population that harbored the diversity of 43 *Ae. tauschii* accessions (**Figure 1**). The production of this population was described by Tsujimoto et al. (2015) and Gorafi et al. (2018). Briefly, it was derived from BC_1_F_1_ plants developed by backcrossing the Japanese wheat cultivar ‘Norin 61’ (N61) to the F_1_ plants from crosses between N61 and 43 different primary synthetic hexaploid wheat lines. The synthetics were produced by crosses between durum wheat (*Triticum durum*, 2n = 28, AABB) cv. ‘Langdon’ and various accessions of *Ae. tauschii* (2n = 14, DD) (**Figure 1**). By self-pollinating the BC_1_F_1_ plants, we obtained 43 sets of BC_1_F_2_ seeds. We took 10 seeds from every 10 BC_1_F_2_ plants and mixed them to obtain a population of 4300 seeds. We grew plants from these BC_1_F_2_ seeds, harvested their BC_1_F_3_ seeds in bulk, randomly selected 3000 seeds and used them to identify genotypes with long dormancy (**Figure 1**). We also used the following materials as check lines or cultivars: common wheat lines OS38, OS108, OS21-5, and OW104, and cultivar ‘Gifu-komugi’, which all have considerable dormancy (Osanai et al., 2005; Kashiwakura et al., 2016); N61, ‘Kitakei-1354’, and ‘Chinese Spring’ (CS), which all have low dormancy.

### Backcross inbred lines

To identify QTLs and candidate genes underlying seed dormancy in the dormant lines derived from the MSD population, we developed a population of backcross inbred lines (BILs) consisting of 166 genotypes from a cross between N61 and synthetics derived from *Ae. tauschii* accession KU-2039 using single-seed descent (**Figure 1**).

### Screening for seed dormancy and dormant genotype identification in MSD population

We harvested the BC_1_F_3_ seeds of the MSD population in bulk on June 3, 2014, and stored them at 25°C. On July 30 (57 days after harvest), August 6 (64 days after harvest), and August 20 (78 days after harvest), we sowed 1000 grains in a tray with wet filter paper (first screening). After 7 days, the ungerminated seeds at 57, 64, and 78 days after harvest were collected, dried, and stored for the second germination test. On November 4 (154 days after harvest), we sowed the ungerminated seeds from the first screening in a tray with wet filter paper (second screening). We selected the germinated seeds, grew plants, self-pollinated them, and harvested BC_1_F_4_ seeds. In summer 2015, we performed another germination test as described above and selected BC_1_F_4_ plants that showed apparent dormancy.

We isolated DNA from three representative plants of each of the 17 dormant BC_1_F_4_ lines and genotyped them using DArTseq markers to determine which *Ae. tauschii* accessions had contributed the dormancy genes. DNA of all genotypes was extracted following a modified CTAB method (Saghai-Maroof et al., 1984). DNA samples (20 μl; 50–100 ng μL^−1^) were sent to Diversity Array Technology (DArT) Pty., Ltd., Australia3 for whole-genome scanning with the DArTseq (DArT sequencing) platform.

### Intensity of seed dormancy in selected MSD lines

To determine the difference of the dormancy intensity of the selected genotypes, we recorded the flowering date in 2016 spring and obtained BC_1_F_5_ seeds. We performed the germination test at 50-, 90-, and 150-days post anthesis (DPA) at 20°C as described above.

To test the possibility of dormancy breakage by external factors, using seeds at 50 DPA, we examined dormancy breaking by cold water imbibition at 10 and 15°C compared with the control (20°C).

### Evaluation of seed dormancy in BILs

The BILs (consisted of 166 genotypes) together with three checks (the recurrent parent, the synthetic donor and a black-seeded wheat) were planted for two seasons, S1 (2019/2020) and S2 (2020/2021), in the field of the Arid Land Research Center (ALRC), Tottori University, Tottori, Japan (35°32′ N, 134°13′ E, 11 m a.s.l.). In each season, two plants from each genotype were grown in an augmented randomized complete block design with the three checks replicated in five blocks. At physiological maturity, 10 spikes per genotype were harvested, air-dried for 10 days in a greenhouse, and then stored at 25°C for 90 days after harvesting. Spikes were hand-threshed, and 20 seeds per genotype were used for the germination test in plastic trays (35 × 25 cm and 4.5 cm depth). Seeds were placed on three layers of moistened tissue paper (250 ml of water) and kept at 24–24.5°C for 7 days. Germinated seeds were counted and removed daily until the end of the test. In each season, the germination percentage was calculated as:


$$G\left(\%\right)=\frac{\sum_{i=1}^{k}n_{i}}{N}\;\times 100,$$

where *n_i_* is the number of seeds germinated at the *ith* time point and *N* is the total number of seeds.

The germination index was calculated by following the formula of Coolbear et al. (1984):


$$GI=\;\sum_{i=1}^{k}n_{i}/t_{i},\;$$

where *t_i_* is the time taken for seeds to germinate at the *i^th^* time point.

### Phenotypic data analysis

We performed ANOVA on the data of G% and GI for each season. Then, the combined analysis was performed considering genotype, season and their interaction as random effects using Plant Breeding Tools V.1.4.2 software. The best linear unbiased prediction (BLUP) means for G% and GI were used for the QTL analysis.

### Genotyping of the BILs population, map construction, and QTL analysis

Total genomic DNA was extracted from 2-week-old leaves of the BILs using the CTAB method (Saghai-Maroof et al., 1984). We sent the DNA samples (20 µL; 50–100 ng µL^−1^) to Eurofins Genomics Company, Japan (https://eurofinsgenomics.jp/jp/home/) for a whole-genome scan with Genotyping by Random Amplicon Sequencing-Direct (GRAS-Di) markers.

The 166 BILs were genotyped with 21,555 GRAS-Di markers. We removed markers amplified in all samples from all parents, markers of low quality (E), and markers with at least one mismatch. The remaining 6,815 markers were used to construct a linkage map. In the first step, we implemented the BIN tool algorithm in the IciMapping software version 4.2 (Meng et al., 2015). The 6,815 markers were binned according to their segregation pattern. After binning, we grouped the markers using a logarithm of odds (LOD) threshold value of 3.0. Linkage groups were assigned according to the genomic position of the SNP markers determined during SNP calling. Recombination frequencies between markers were converted into centiMorgans (cM) using the Kosambi mapping function. We used the R/qtl (Arends et al., 2010) packages available in the R Statistical Computing Environment to inspect the initial linkage map for duplicate lines, segregation distortion, switched alleles, and single and double cross-overs (genotyping errors). Lastly, after removing low-quality markers and correcting the genotyping error, the genotypic data of 166 BIL lines with 2,882 high-quality markers were used to construct the final genetic map in IciMapping 4.2.

The values of G% and GI of each season separately and combined for the 166 BILs were used for QTL mapping in QTL IciMapping 4.2 software. Inclusive composite interval mapping of QTL with additive and dominance effect (ICIM-ADD) analysis was conducted using G% and GI phenotypic data of the 166 BIL lines with 2882 molecular markers. The significant LOD threshold (3.0) for declaring a QTL (α = 0.05) was determined from 10,000 permutations. The R software package R/qtl was used to draw the LOD curves of the QTLs.

### GWAS analysis

The germination percentage and index for the two seasons and their combined for the 166 BILs were used for the GWAS analysis using the 2,882 GRAS-Di markers. The GAPIT package (Lipka et al., 2012) in R software V.4.2.2 was used to perform genome-wide association analyses (GWAS) using a fixed and random model with circulating probability unification (FarmCPU) (Neves et al., 2012). FarmCPU provides more statistical power than the general linear model (GLM) and mixed linear model (MLM) and also decreases confounding effects. To prevent overfitting, a random-effect model is used to select associated markers using a maximum likelihood method, while a fixed-effect model is used to test the remaining markers using iteratively detected associated markers as cofactors. The results of GWAs were visualized using Manhattan plots (Turner, 2018).

### Candidate gene analysis

To verify the physical positions of QTL flanking markers, we blasted the flanking markers against the CS reference genome sequence (RefSeq v2.1) published by the International Wheat Genome Sequencing Consortium (IWGSC; https://wheat-urgi.versailles.inra.fr/; accessed in March 2023) and extracted the genes located between the flanking markers with100% confidence for the genome region. We investigated the expression levels of the candidate genes and compared them to the expression of the known dormancy gene *TaMFT* as a reference (Nakamura et al., 2011) using the Expression Atlas Browser (https://www.ebi.ac.uk/gxa/home) (Papatheodorou et al., 2020).

## Results

### Identification of dormant lines in MSD population

We conducted the first screening using the BC_1_F_3_ seeds of the MSD population (**Figure 1**). Three germination tests were performed. The first test (test A) was performed at 57 days after harvest (DAH), whereas the second (test B) and the third (test C) tests were performed at 64 and 78 DAH, respectively. Out of 1,000 seeds sown at test A, test B, and test C, only 29, 67, and 18 seeds were not germinated, respectively, while all N61 seeds germinated. In the second screening, we sowed these 114 ungerminated seeds 154 DAH, and 80 seeds (70.2%) germinated. We randomly selected 17 dormant lines out of the 80 germinated seeds, advanced them to BC_1_F_4_ and established 17 dormant lines.

To determine which *Ae. tauschii* accessions contributed dormancy to these selected BC_1_F_4_ plants, we isolated DNA from three representative BC_1_F_4_ plants from each of the 17 lines and genotyped them using DArTseq markers. We found that dormant plants originated from 9 of the 43 *Ae. tauschii* accessions: KU-2039, PI476874, KU-20-9, KU-2092, KU-2093, KU-2124, KU-2156, AT55, and AT76 (**Supplementary Table 1**). KU-2039 and PI476874 from lineage 1 (TauL1) were collected in Afghanistan; both belong to the variety *typica* (Mahjoob et al., 2021) and are phylogenetically very similar. KU-20-9, KU-2092, KU-2093, KU-2124, and KU-2156 are all from Iran and belong to TauL2. AT55 and AT76 are from China, have a similar phylogenetic history and belong to TauL1.

The genetic analysis of the 17 dormant MSD lines revealed that eight lines were found to be originated from KU-2039 (Syn32), two lines from PI476874 (Syn49) and KU-2092 (Syn37). The other five *Ae tauchii* accessions contributed by only one MSD line each (**Supplementary Table 1**).

For further analysis of seed dormancy intensity, we selected a total of eight lines, five MSD (126, 130, 132, 142, and 143) derived from KU-2039 and three MSD (129-1, 129-2 and 129-4) from KU-2124 in addition to the eight checks.

### Intensity of seed dormancy in selected MSD lines

**Figure 2:**
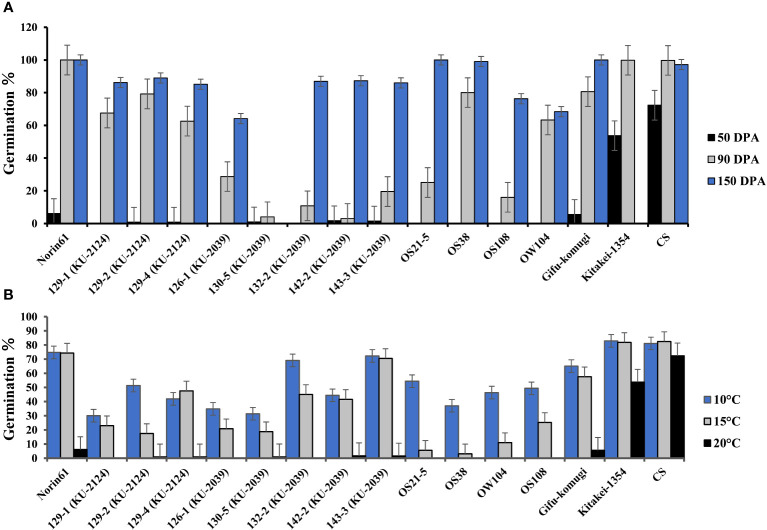
Germination rates of ‘Norin 61’, lines selected from the MSD population, and some wheat lines. The MSD lines were derived from *Aegilops tauschii* accessions KU-2039 or KU-2124 as indicated. **(A)** Seed dormancy; **(B)** disruption of seed dormancy after 12 days of cold-water treatment (10°C, 15°C, or 20°C). Error bars indicate standard error.

At 50 days past anthesis (50 DPA), the germination % (G%) of the BC_1_F_5_ MSD lines derived from KU-2039 or KU-2124 ranged from 0-4%, whereas N61 recorded 6%. No germination was found for the four dormant checks; however, the range of G% was 6-72% for the non-dormant checks (**Figure 2A**). At 90 DPA, the MSD lines derived from KU-2039 showed the lowest germination range, followed by lines derived from KU-2124. The recurrent parent, N61, recorded 100% germination, whereas the G% of the dormant checks ranged 16-80%. At 150 DPA, most of the lines derived from both KU-2039 and KU-2124 showed germination higher than 80%, except MSD126-1, which recorded comparable G% with that of OS108 and OW104.

After cold water imbibition, germination percentage increased with decreasing water temperature with varying degrees among the MSD lines and dormant checks. Most of the lines from KU-2039 and KU-2124 showed similar or lower G% at 10°C compared to the dormant checks (**Figure 2B**).

### Evaluation of dormancy in KU-2039 BILs

We developed 166 BILs from crossing and backcrossing N61 with a synthetic wheat line (Syn32) originated from KU-2039. To identify the best timing for seed dormancy evaluation in the BILs population, we carried out germination test for the two parents and Syn44 at different times from harvest. After 90 days from harvest (90DAH), the G% of Syn32 was 28%, whereas N61 and Syn44 recorded 100 and 97% germination, respectively (**Supplementary Figure 1**). Therefore, 90DAH was selected to be used for the evaluation of G% and GI of the BILs.

**Figure 3:**
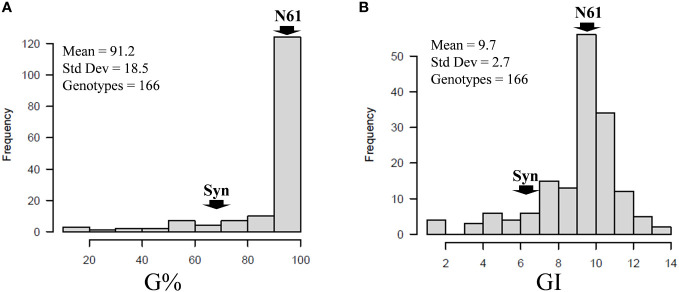
Frequency distributions of **(A)** germination percentage (G%) and **(B)** germination index (GI) in the population of 166 BILs. Arrows indicate the parental lines, Norin 61 (N61) and synthetics (Syn) derived from KU-2039. The mean, standard deviation, and the number of genotypes indicated in each panel.

Combined across the two seasons (S1 and S2), 25 BILs, out of the 166 BILs, showed lower G% than that of the Syn32 (75%), whereas the recurrent parent, N61, recorded 100% germination (**Figure 3**; **Supplementary Table 2**). Similarly, 26 BILs showed lower GI than that of the Syn32 (6.8), whereas N61 recorded GI of 11.5 (**Figure 3**; **Supplementary Table 2**). The G% over the two seasons in the BILs and their parents 90DAH revealed that 28 BILs showed G% < 50 at least in one of both seasons. Out of the 28 BILs, five lines, consistently showed G% < 50 at both seasons (**Table 1**; **Supplementary Table 2**). N61 recorded 100% germination in both seasons, whereas Syn32 showed 50% in SI and 100% in S2. The GI for N61 and Syn32 were 9.5 and 1.7 in S1, 13.5 and 12.0 in S2, respectively (**Table 1**; **Supplementary Table 2**).

**Table 1:** Germination percentage (G%) and germination index (GI) of the dormant lines selected from the 166 BILs tested for two seasons (S1 and S2).

Genotypes	G%			GI		
	S1	S2	Combined	S1	S2	Combined
N61(Recurrent)	100.0	100.0	95.3	9.5	13.5	10.9
Syn32 (Donor)	50.0	100.0	83.5	1.7	12.0	7.8
BW	100.0	100.0	95.3	11.5	15.0	12.0
BIL1	0.0	93.3	70.2	0.0	9.3	6.4
BIL3	26.7	46.7	65.5	1.1	5.5	5.5
BIL10	53.3	86.7	81.1	2.3	10.0	7.4
BIL18	20.0	100.0	76.4	1.8	13.5	8.4
BIL20	6.7	86.7	70.2	0.1	10.5	6.8
BIL25	26.7	86.7	74.9	1.1	8.7	6.6
BIL32	93.3	0.0	70.2	8.3	0.0	6.1
BIL46	100.0	0.0	71.7	7.0	0.0	5.7
BIL48	40.0	20.0	62.3	1.6	1.5	4.4
BIL49	53.3	73.3	78.0	2.9	7.5	6.8
BIL54	40.0	53.3	70.2	1.8	8.0	6.6
BIL77	60.0	100.0	85.9	2.7	13.0	8.5
BIL81	33.3	93.3	78.0	1.6	9.5	7.0
BIL85	13.3	26.7	57.6	0.7	2.5	4.4
BIL86	0.0	20.0	52.9	0.0	2.5	4.2
BIL88	86.7	13.3	71.7	6.8	1.5	6.1
BIL94	86.7	53.3	81.1	7.3	5.0	7.4
BIL127	86.7	46.7	79.6	3.9	5.0	6.3
BIL128	93.3	40.0	79.6	5.0	3.5	6.1
BIL129	40.0	53.3	70.2	1.8	4.5	5.4
BIL139	100.0	33.3	79.6	6.3	2.8	6.3
BIL141	100.0	46.7	82.7	5.9	4.5	6.8
BIL164	13.3	20.0	56.1	1.3	2.0	4.5
Mean			91.2			9.7
SD			16.7			2.3
*P-*value			<0.001			<0.001
Heritability			0.47			0.65

BW, black wheat.

The overall mean, standard deviation (SD), and the probability (*P-value*) of the combined analysis are also included.

### Genetic mapping of the dormancy trait

**Figure 4:**
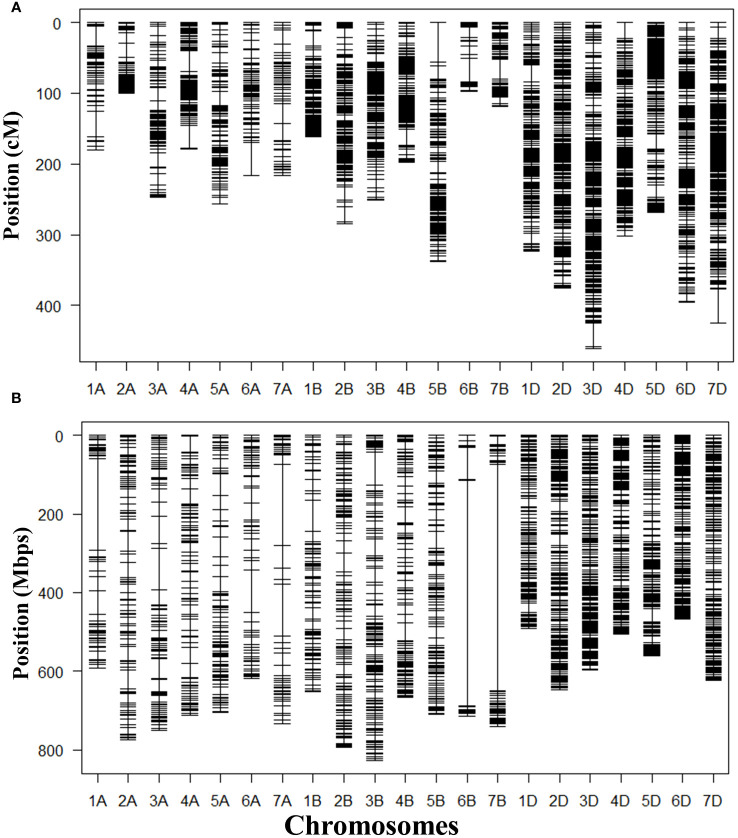
Maps constructed with 2,882 GRAS-Di markers mapped in the population of 166 BILs derived from a cross between N61 and a synthetic wheat line derived from *Aegilops tauschii* accession KU-2039. **(A)** Genetic map; **(B)** physical map.

GRAS-Di genotyping used 21,555 markers, of which 6,815 were polymorphic between the synthetic wheat donor parent derived from KU-2039 and the backcross parent N61. Among the polymorphic markers, 2,882 (42.3%) were of high quality, with an average of 137 markers on each chromosome (**Supplementary Figures 2–5**). A linkage map constructed with the 2,882 markers distributed over 21 linkage groups covered a genetic distance of 5,528.90 cM (**Table 2**) with an average of 263.28 cM per chromosome. Markers were unevenly distributed across chromosomes and sub-genomes. Most markers (1,562; 54.2%) were mapped to the D sub-genome, with a total genetic length of 2,550.77 cM, while 741 (25.7%) markers were mapped to the B sub-genome with a total genetic length of 1,449.55 cM. The lowest number of markers (579; 20.1%) with a total genetic length of 1,528.59 cM were mapped to the A sub-genome (**Figure 4A**). In terms of genetic map length, the D sub-genome was the longest, followed by the A and B sub-genomes. The D sub-genome had the highest marker density (one marker per 1.63 cM), followed by the B (one marker per 1.96 cM) and A sub-genomes (one marker per 2.64 cM) (**Table 3**). The highest number of markers (276) was on chromosome 3D, with a genetic distance of 460.61 cM, and the lowest (23 markers) was on chromosome 6B, with a genetic distance of 96.94 cM. Seven gaps greater than 30 cM were found on chromosomes 1A, 3D, 4A, 5B, 6A, 6B, and 7D (**Supplementary Table 3**).

**Table 2:** Description of basic characteristics of the 21 chromosomes with their genetic and physical distance.

Chromosome	No. of markers	Chromosome length (cM)	Average distance between markers (cM)	Chromosome length (Mb)	Average distance between markers (Mb)
1A	51	180.65	3.61	591.38	11.87
2A	105	231.72	2.22	774.27	7.44
3A	90	247.39	2.77	748.88	8.41
4A	110	179.25	1.79	711.39	7.11
5A	103	256.54	2.51	704.37	6.90
6A	70	216.42	3.13	618.23	8.95
7A	50	216.61	4.42	732.41	14.90
1B	101	162.03	1.62	652.67	6.52
2B	150	284.98	1.91	792.61	5.31
3B	146	251.39	1.73	825.90	5.69
4B	121	197.56	1.64	665.15	5.45
5B	141	337.99	2.41	709.75	5.06
6B	23	96.94	4.40	712.88	6.68
7B	59	118.67	2.04	740.06	12.70
1D	188	323.33	1.72	491.40	2.62
2D	250	375.97	1.50	647.61	2.60
3D	276	460.61	1.67	597.00	2.17
4D	194	301.36	1.56	505.70	2.60
5D	174	268.54	1.55	560.44	3.23
6D	219	395.63	1.81	472.40	2.10
7D	261	425.34	1.63	622.20	2.39
Total	2882	5528.90	2.26	13876.69	6.22

**Table 3:** Description of the basic characteristics of the three sub-genomes, A, B, and D.

A sub-genome			B sub-genome			D sub-genome		
Chromosomes	Chromosome length	Marker number	Chromosomes	Chromosome length	Marker number	Chromosomes	Chromosome length	Marker number
1A	180.65	51	1B	162.03	101	1D	323.33	188
2A	231.72	105	2B	284.98	150	2D	375.97	250
3A	247.39	90	3B	251.39	146	3D	460.61	276
4A	179.25	110	4B	197.56	121	4D	301.36	194
5A	256.54	103	5B	337.99	141	5D	268.54	174
6A	216.42	70	6B	96.94	23	6D	395.63	219
7A	216.61	50	7B	118.67	59	7D	425.34	261
Total	1528.58	579		1449.55	741		2550.77	1562
Average	218.40	82.7		207.10	105.9		364.40	223.1
% in genome	27.6	20.1		26.2	25.7		46.1	54.2
Marker density	2.64			1.96			1.63	

We constructed a physical map that spans 13,876.69 Mb using the marker positions acquired from GRAS-Di (Eurofins Genomics) based on the N61 reference genome. The longest chromosomes were 3B (825.90 Mb) and 2B (792.60 Mb), and the shortest was 6D (472.41 Mb). The average physical distance between markers was 1.40 Mb. The length of the A sub-genome was 4,880.93 Mb, that of the B sub-genome was 5,099.01 Mb, and that of the D sub-genome was 3,896.75 Mb (**Table 2**). In general, markers were denser in the telomeric regions than in the centromeric regions (**Figure 4B**). To validate the suitability of this map for QTL analysis, we performed a QTL analysis for the trait with a well-known QTL position (i.e., days to heading). The QTL for days to heading was found on chromosome 2D at 173.21 cM, which perfectly matched the position of *Ppd-D1* (photoperiod-responsive gene) (**Supplementary Figure 6**) (Hanocq et al., 2004).

### QTL detection

In the first season, the ICIM-ADD detected one QTL on chromosome 5D at 72 cM (LOD of 10.34) associated with G% with flanking markers AMP0017090 (at 70.86 cM) and AMP0004316 (at 72.26 cM), explaining 26.47% of the phenotypic variance, while no QTL associated with GI was detected (**Table 4**; **Supplementary Figures 7A, B**; **Supplementary Table 3**).

**Table 4:** QTLs detected by Inclusive composite interval mapping with additive and dominance effect (ICIM-ADD) analysis.

Season	Trait	QTLs	Chromosome	Position (cM)	Left Marker	Right Marker	LOD	PVE (%)	Add
S1	G%	*QSd.arlc-5D*	5D	72	AMP0017090	AMP0004316	10.3436	26.4674	14.494
S2	G%		1D	134	AMP0000577	AMP0024860	4.0755	3.1939	5.5217
		*Qsd.alrc.5D1*	5D	43	AMP0013081	AMP0024991	29.2586	33.5783	17.8987
		*QSd.arlc-5D2*	5D	49	AMP0031012	AMP0032707	12.677	11.3921	-10.42
		*QSd.arlc-5D*	5D	74	AMP0020940	AMP0025926	17.1079	15.9853	11.5551
	GI		5B	257	AMP0005269	AMP0036949	3.5827	7.9631	0.811
		*QSd.arlc-5D3*	5D	33	AMP0027328	AMP0032656	6.8755	16.3094	1.3658
		*QSd.arlc-5D*	5D	74	AMP0020940	AMP0025926	12.4475	31.449	1.7951
combined	G%	*QSd.arlc-5D*	5D	72	AMP0017090	AMP0004316	19.0615	41.9494	15.5069
	GI		5D	72	AMP0017090	AMP0004316	11.6362	27.8412	1.4874

LOD, logarithm of odds; PVE, phenotypic variance explained; ADD, additive effect.

In the second season, 4 QTLs were detected, one on chromosome 1D at 134 cM (LOD of 4. 07) flanked by AMP0000577 (at 133.46 cM) and AMP0024860 (at 136.98 cM), explaining 3.23% of the phenotypic variance of G%, and three on chromosome 5D at 74 cM (LOD of 17.10) near to the QTL identified in the first season, 43 cM (LOD of 29.26) and 49 cM (LOD of 12.68), with flanking markers AMP0020940 (at 72.72 cM) and AMP0025926 (at 74.10 cM), AMP0013081 (at 42.69 cM) and AMP0024991 (at 43.17 cM), AMP0031012 (at 47.94 cM) and AMP0032707 (at 49.40), explaining 15.98, 33.78, and 11.40% of the phenotypic variance of G%, respectively (**Table 4**; **Supplementary Figures 7C–E**; **Supplementary Table 3**). Three QTLs were associated with GI, one on chromosome 5B at 257 cM (LOD of 3.58) flanked by AMP0005269 (at 256.29 cM) and AMP0036949 (at 257.11 cM), explaining 7.96% of the phenotypic variance, the other two were in chromosome 5D at 74 cM (LOD of 12.45) near to the QTLs identified for G% in the two seasons, and at 33 cM (LOD 6.87) with flanking markers AMP0020940 (at 72.72 cM) and AMP0025926 (at 74.10 cM), and AMP0027328 (at 32.70 cM) and AMP0032656 (at 33.65 cM), explaining 31.45 and 16.31% and of the phenotypic variance, respectively (**Table 4**; **Supplementary Figures 8A–C**; **Supplementary Table 3**).

**Figure 5:**
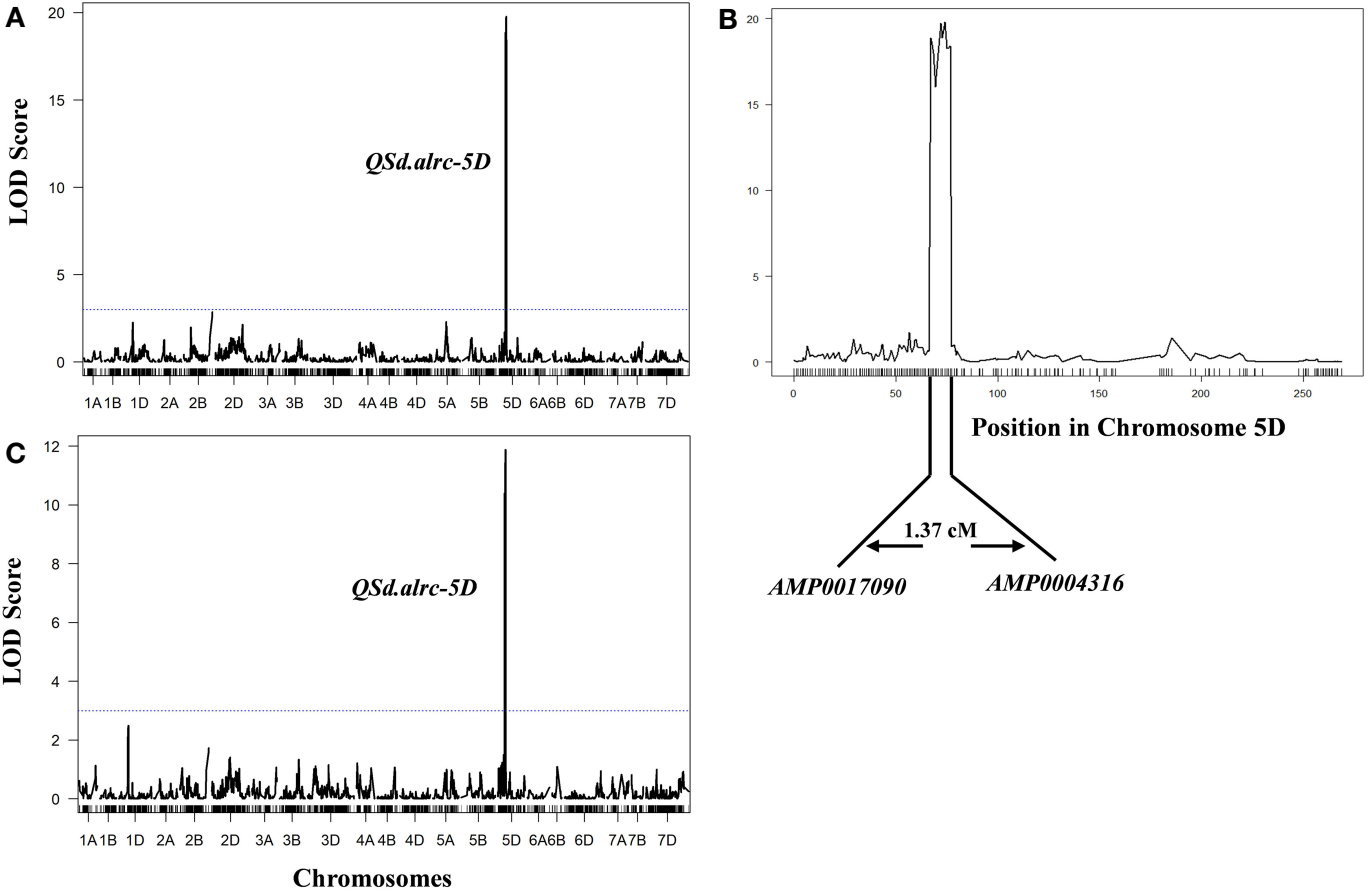
Mapping of QTLs for germination percentage and index with the ICIM-ADD method. **(A)** The identified QTL for germination percentage; **(B)** the specific position of the QTL on chromosome 5D with the flanking markers; **(C)** the identified QTL for germination index. The dash-dotted blue lines indicate the threshold of the LOD score at 3.0.

The combined G% and GI for the two seasons revealed one major QTL underlying seed dormancy in the BILs at 72 cM on chromosome 5D associated with G% or GI, with the flanking markers AMP0017090 (at 70.89 cM) and AMP0004316 (at 72.26 cM), explained 41.94% (LOD of 19.06) and 27.84% (LOD of 11.63) of the phenotypic variance of G% and GI, respectively (**Figure 5**; **Table 4**). We named this major QTL *Qsd.alrc.5D* and the other QTLs identified on chromosome 5D in the second season *Qsd.alrc.5D1, Qsd.alrc.5D2, and Qsd.alrc.5D3* (**Table 4**) following the guidelines for gene nomenclature in wheat (Boden et al., 2023).

We conducted GWAS using the physical map positions to verify the results of QTL analysis and to confirm the physical position of the QTL. GWAS detected highly significant marker-trait association on chromosome 5D at 339.59 Mbp (correspond to 72.72 cM) associated with G% or GI in S1, S2 and their combined confirming the results of the QTL analysis and suggesting that the *Qsd.alrc.5D* identified in the first and second season in different but very close positions and in the combined analysis is the same QTL. On the other hand, GWAS further confirmed the results of the QTL analysis and detected the QTLs identified on chromosomes 5B, 1D and 5D identified in the second season (**Supplementary Figures 9, 10**).

### Identification of candidate genes

**Figure 6:**
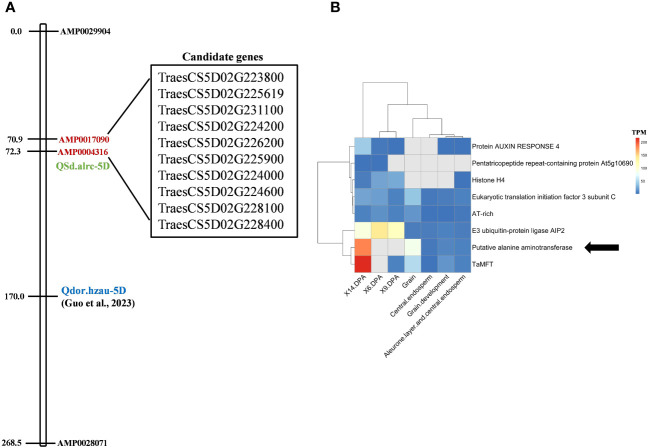
Candidate dormancy genes located between the *Qsd.alrc-5D*-flanking markers. **(A)** Candidate genes with known function; **(B)** heat map of the expression of the likely (associated with ABA regulation or dormancy) candidate genes and the well-known reference dormancy gene *TaMFT*. The arrow indicates the most likely candidate gene associated with dormancy. *Qdor.hzau-5D*, a minor QTL for seed dormancy; X6.DPA, grain development stage at 6-day post anthesis; X9.DPA, grain development stage at 9-day post anthesis; X14.DPA, grain development stage at 14-day post anthesisTables.

In the CS reference genome, 76 genes are located between the flanking markers of *Qsd.alrc-5D*, and 10 have well-identified functions (**Supplementary Table 4**; **Figure 6A**). Of the 10 genes, 7 have functions related to seed dormancy or abscisic acid (ABA) (**Table 5**). An analysis of gene expression patterns of the seven genes revealed a possible association between *TraesCS5D02G224200*, which encodes alanine aminotransferase, and the reference dormancy gene *TaMFT* (**Figure 6B**).

**Table 5:** Identified candidate genes found between the franking markers of *Qsd.alrc-5D*.

No	Genes	Chr	Position (bp)	Description	Functions
**1**	*TraesCS5D02G223800*	5D	331,572,260 - 331,575,937	E3 ubiquitin-protein ligase *AIP2*	Negative regulator of ABA signaling
**2**	*TraesCS5D02G225619*	5D	334,000,352 - 334,021,279	Pentatricopeptide repeat-containing protein At5g10690	ABA regulation
**3**	*TraesCS5D02G231100*	5D	338,386,897 - 338,390,472	Phospholipase D	Mediate plant responses to abscisic acid (ABA)
**4**	*TraesCS5D02G224200*	5D	332,040,151- 332,044,580	Putative alanine aminotransferase	Controlling dormancy
**5**	*TraesCS5D02G226200*	5D	334,320,783 -334,323,090	Protein AUXIN RESPONSE 4	Seed dormancy control
**6**	*TraesCS5D02G225900*	5D	334,257,035 - 334,291,837	Glutamate receptor	Regulating Seed germination under salt
**7**	*TraesCS5D02G224000*	5D	331,841,530 - 331,842,234	Histone H4	Histone modifications mediate seed dormancy and germination

## Discussion

### QTL and candidate gene for seed dormancy derived from *Ae. tauschii*

Seed dormancy is one of the most important traits that plays a critical role in the resistance of wheat grain to PHS, especially in wheat production areas where frequent rain and high humidity are combined with cool temperatures at harvest time (Singh et al., 2021). In this study, we screened 3,000 seeds from the MSD population, isolated genotypes with strong seed dormancy, and revealed that they originated from only 9 accessions of *Ae. tauschii* out of 43 accessions used to develop the MSD population (**Supplementary Table 1**). Of these nine accessions, those from Afghanistan tended to provide longer dormancy than those from Iran and China. Afghanistan is an arid region, and it is unlikely that *Ae. tauschii* in this region has acquired PHS tolerance as a result of environmental adaptation. This suggests that the gene was selected because it confers tolerance to other factors, such as hot and dry summers or cold winters, and that the mechanism of seed dormancy may be linked to such traits at the molecular level.

In this study we constructed a linkage map to detect the QTLs associated with seed dormancy. The linkage map, distributed over 21 linkage groups, covered a genetic distance of 5,528.90 cM with an average of 263.28 cM per chromosome. The length of our map is comparable to those for wheat, e.g. 5,257 cM reported by Cui et al. (2014) and 5,332 cM reported by Röder et al. (1998) (for a review, see Langridge et al. (2001). In this study, the D sub-genome was the longest (2,550.77 cM), with the highest number of markers (1,562) and the highest marker density (1.63 cM per marker), followed by the A and B sub-genomes (**Table 3**). The higher level of polymorphism in the D sub-genome can be explained by its origin from the wild D genome introduced from *Ae. tauschii*. Most of the previous genetic maps of wheat reported the D sub-genome as the shortest with the lowest number of markers (Hussain et al., 2017) because of the low level of polymorphism in the D sub-genome (Wang et al., 2014). Thus, this BILs population provides a unique opportunity to study the D sub-genome and could be a valuable breeding material for the development of elite wheat cultivars.

The *Qsd.alrc-5D* was identified as an important novel seed dormancy QTL in this study. *Qsd.alrc-5D* was detected in S1 and S2 in chromosome 5D with a slight shift in the position from 72 in S1 to 74 in S2, and it was also detected in the combined G% and GI at 72 cM. The GWAS revealed that *Qsd.alrc-5D* is the same QTL at 339.59 Mbp (correspond to 72.72 cM). The shift in position of *Qsd.alrc-5D* between S1 and S2 can be explained by considering the degree of gene penetrance: genetic differences in seed dormancy can only be detected at a certain time after harvest. During this period, some carriers of an important dormancy gene may germinate (**Supplementary Figure 1**). On the other hand, the temperature fluctuations during grain development can affect seed dormancy (Mares et al., 2021). The mean temperature in the S1 was 3.2°C lower than in S2 (**Supplementary Figure 12**). Four other QTLs were detected only in S2 on chromosome 1D, 5B, and 5D. These two unstable QTLs on chromosome 1D, 5B with minor LODs have been reported earlier (Liton et al., 2021; Guo et al., 2023). The QTLs *Qsd.alrc-5D2* and *Qsd.alrc-5D3*, detected on chromosome 5D, were not reported, indicating that they might be novel QTLs for seed dormancy. However, further validation is needed. The combined frequency distribution of G% and GI revealed that, the BILs were generally skewed with the peak located over the lower dormancy portion of the distribution (**Figures 3A, B**), indicating the involvement of a major QTL (*Qsd.alrc.5D)* and multiple minor alleles or QTLs (*Qsd.alrc.1D*, *Qsd.alrc.5B*, *Qsd.alrc.5D2* and *Qsd.alrc.5D3*) in controlling seed dormancy. Liton et al. (2021) and others reported a contribution of major and several minor genes or QTLs in controlling PHS or dormancy.

In wheat, QTLs associated with PHS, or seed dormancy have been mapped on multiple chromosomes. QTLs for PHS were identified on chromosomes 4A (Torada et al., 2016), 3A (Nakamura et al., 2011), 2A (Zhang et al., 2017), and 2B (Feng et al., 2019). While for seed dormancy were identified on chromosomes 4A (Kumar et al., 2015; Liton et al., 2021) 3A (Shao et al., 2018) 2B (Kumar et al., 2009), 3B (Mares et al., 2009), 4B (Kato et al., 2001), 3D (Imtiaz et al., 2008), and 4D (Kato et al., 2001). Guo et al. (2023) reported a minor QTL for seed dormancy on chromosome 5D (*Qdor.hzau-5D*) at 170 cM with a LOD value of 4.68. Here, we identified a significant major QTL for seed dormancy on chromosome 5D at 72 cM, corresponding to 339.59 Mbp, with an LOD value of 19 and explained 41.7% of the phenotypic variation (**Figure 5**). Considering that *Qdor.hzau-5D* is located at a different locus (97.7 cM away from our QTL; **Figure 6A**; **Supplementary Figure 11**) and that it is minor, our *QSd.alrc-5D* is obviously a novel and major QTL associated with seed dormancy.

### Causal gene for seed dormancy

The publicly available genomic sequence of CS (IWGSC 2023) can be used to identify positional candidate genes underlying QTLs in the vicinity of markers. We attempted to identify positional and functional candidate genes underlying *QSd.alrc-5D* between the significant flanking markers AMP0017090 and AMP0004316 (332.706225–339.593142 Mbp). We searched for possible candidate genes associated with seed dormancy or ABA in this region. ABA is a plant stress response hormone that plays a critical role in regulating seed dormancy (Tuan et al., 2018). Grain ABA levels, ABA biosynthesis, catabolism, or both, and seed sensitivity to ABA are regulated by the expression of genes involved in ABA signaling (Barrero et al., 2012) and are important in regulating dormancy.

The *QSd.alrc-5D* region contained 76 putative genes (**Supplementary Table 4**). Among them, *TraesCS5D02G231100*, located between 338.386897 and 338.390472 Mb, encodes phospholipase D. The phospholipase family mediates responses to ABA (Hong et al., 2016) in Arabidopsis (Zheng et al., 2012), rice (Singh et al., 2013), and wheat (Khalil et al., 2011). *TraesCS5D02G223800*, located between 331.57226 and 331.575937 Mb, encodes the E3 ubiquitin-protein ligase *AIP2*, which functions as a negative regulator of ABA signaling by polyubiquitinating ABI3 and presumably targeting it to the 26S proteasomes for degradation (Gao et al., 2014). *TraesCS5D02G226200*, located between 334.320783 and 334.323090 Mb, encodes protein auxin response 4. Auxin regulates seed dormancy in Arabidopsis by stimulating ABA signaling through ARF-mediated *ABI3* activation and delays seed germination in wheat (Liu et al., 2013). *TraesCS5D02G226200*, located between 331.841530 and 331.842234 Mb, encodes histone H4. Histone regulates ABA, and its modification are reportedly the major mechanisms of seed dormancy and germination (Zhao et al., 2019). *TraesCS5D02G225619*, located between 334.000352 and 334.021279 Mb, encodes a pentatricopeptide repeat (PPR)–containing protein. PPR proteins are involved in ABA signaling and ABA sensitivity under biotic and abiotic stresses (Li et al., 2021). *TraesCS5D02G225900*, located between 334.257035 and 334.291837 Mb, encodes a glutamate receptor (GLR). GLR3.5 stimulates seed germination by antagonizing the inhibition of ABA (Kong et al., 2015). Since ABA levels and ABA signaling play crucial roles in regulating seed dormancy and germination, these six genes might regulate seed dormancy associated with *QSd.alrc-5D*. In gene expression analysis, *TraesCS5D02G224200*, located between 332.040151 and 332.044580 Mb and encoding putative alanine aminotransferase (AlaAT), clustered with the reference seed dormancy gene *TaMFT* and was highly expressed at 14 days post anthesis of grain development (**Figure 6B**). AlaAT is reportedly the causal protein for the barley grain dormancy QTL, *Qsd*1 (Sato et al., 2016). AlaAT plays a critical role in gluconeogenesis, the metabolic process that generates glucose from non-carbohydrate carbon substrates. This process is linked to the revival of plastid functions in dormant rice seeds following imbibition (Gianinetti et al., 2018). Therefore, AlaAT is likely the major causal protein for the seed dormancy phenotype associated with *QSd.alrc-5D*. Because the segregation of seed dormancy in our population indicated the presence of a major gene and minor genes, some ABA-related genes may be the minor genes affecting seed dormancy in our population.

### Genetic and physical maps constructed using GRAS-Di markers

The GRAS-Di platform generates a large number of genetic markers distributed across all chromosomes and enables the construction of high-resolution linkage maps (Miki et al., 2020). We constructed a genetic map using 2882 GRAS-Di markers polymorphic between the parent synthetics and N61. The map spanned 5528.9 cM which is relatively long; this could be explained by the high density of GRAS-Di markers, which generally increases the total length of the linkage map (Miki et al., 2020), and by the gaps (>30 cM) in seven chromosomes.

We constructed a physical map with a total length of 13,876.69 Mb. Semagn et al. (2021) constructed physical maps of four spring wheat populations and reported map lengths of up to 13,788 and 13,881 Mb. In the physical map, the B sub-genome was the longest (5099.01 Mb), followed by the A sub-genome (4880.93 Mb) and the D sub-genome (3896.75 Mb). Telomeric regions have reportedly more markers than the centromeric regions (Peleg et al., 2008). This phenomenon is related to the recombination rate in wheat, as >85% of wheat genes are in gene-rich regions, which are predominantly located in telomeres (Qi et al., 2004).

### MSD as a platform for mining *Ae. tauschii* genes

Wheat is one of the most important cereal crops, providing food for millions of people. Breeders and researchers are constantly looking for innovative ways to improve wheat yield and quality. They focus on enriching the diversity of common wheat by introducing desirable traits from wild wheat relatives such as *Ae. tauschii*. The low diversity in wheat elite breeding material, particularly in the D sub-genome, has hampered linkage map construction, QTL detection, marker discovery, and marker-assisted breeding. Therefore, new genetic diversity for essential traits has been introduced into elite cultivars since the introduction of synthetic wheat in the 1980s (Dale et al., 2017). However, in breeding programs, breeders typically focus on synthetics derived from a limited number of *Ae. tauschii* accessions whose choice was based on trait screening in the wild species. This approach is sometimes futile because *Ae. tauschii* traits may not be expressed at the hexaploid level (Sohail et al., 2011). Thus, it is better to evaluate *Ae. tauschii* traits in the hexaploid level, which requires populations harboring high intraspecific diversity of *Ae. tauschii*. In this study, we used a unique MSD population with diversity from 43 different *Ae. tauschii* accessions in the background of N61 (**Figure 1**) (Tsujimoto et al., 2015; Gorafi et al., 2018). We used 3,000 randomly selected MSD seeds to identify dormant genotypes. We genotyped the plants showing seed dormancy to reveal their pedigree, used this pedigree information to generate a segregating population (BILs), and finally identified a major novel QTL underlying seed dormancy derived from *Ae. tauschii*. Of the 43 *Ae. tauschii* accessions used to generate the MSD population, only the offspring of 9 accessions (representing 21%) had seed dormancy. Thus, had we not used this diverse population, we would not have discovered this valuable gene, which is rare within the species. We believe that the approach used in this study is unique as it allowed us to systematically evaluate the seed dormancy of 43 *Ae. tauschii* accessions at the hexaploid level and enabled us to reveal the rare sources of seed dormancy and to identify a novel and important QTL that can be used in wheat breeding through marker-assisted selection after appropriate validation.

Previous studies have used a limited number of MSD lines to identify germplasm lines and marker trait associations for seed characteristics, drought tolerance and bread making quality (Elhadi et al., 2021a; Elhadi et al., 2021b; Itam et al., 2022; Mohamed et al., 2022a; Mohamed et al., 2022b). The platform proposed in this study would be more efficient as it would allow not only the identification of the desired phenotypes but also the identification of the best candidate among the selected lines. Moreover, the biparental QTL mapping using the population developed or advanced only from the selected line/lines would be more powerful than the identification of the marker trait association using a limited number of lines. Thus, the approach (platform) used in this study represents a unique way to mine *Ae. tauschii* genes or alleles necessary for wheat breeding and improvement to overcome challenges of low genetic diversity.

## Conclusion

This study resulted in the discovery of a novel QTL for seed dormancy from *Ae. tauschii.* Using a wheat population harboring genetic diversity from many *Ae. tauschii* accessions, we first selected plants with the dormancy trait, then developed BILs, identified a QTL, and mapped it on chromosome 5D. This rare QTL, named *QSd.alrc-5D*, might have been missed in previous studies because of the use of a small number of *Ae. tauschii* accessions. Overall, this population has the potential to significantly contribute to the advancement of wheat breeding by allowing systematic mining of the *Ae. tauschii* gene pool in the genetic background of hexaploid wheat. After appropriate validation, the use of *QSd.alrc-5D* would contribute to wheat breeding through marker-assisted selection.

## Data availability statement

The raw data supporting the conclusions of this article will be made available by the authors, without undue reservation.

## Author contributions

HT: Conceptualization, Funding acquisition, Project administration, Resources, Supervision, Writing – review & editing. MA: Data curation, Formal Analysis, Investigation, Validation, Visualization, Writing – original draft. YG: Conceptualization, Data curation, Investigation, Methodology, Supervision, Validation, Writing – review & editing. NK: Data curation, Writing – review & editing. MB: Data curation, Writing – review & editing. IT: Writing – review & editing. LZ: Investigation, Writing – review & editing. NK: Supervision, Writing – review & editing.
