# Supplementary material for: Mining Aegilops tauschii genetic diversity in the background of bread wheat revealed a novel QTL for seed dormancy
Source: Front Plant Sci. 2023 Nov 30;14:1270925. doi: 10.3389/fpls.2023.1270925 (PMC10723804; doi:10.3389/fpls.2023.1270925)
Supplement: Supplementary file 1 [file DataSheet_1.docx]

Supplementary Material

**Supplementary Figure 1.** Germination percentage of Norin 61 (N61), synthetic 44 (Syn44), and the synthetic 32 (Syn32) from the MSD population. It is obvious that Syn32 has the deepest dormancy comparing with N61 and Syn44. This figure showed the difficulty to identify a point that can separate the dormant from the non-dormant. The gray shaded area indicated the time we selected to set our germination experiment.

**Supplementary Figure 2.** Frequency distribution of the number of recombinant inbred lines sharing the same genotypic.

**Supplementary Figure 3.** Pairwise recombination fractions of all marker pairs. An increase in recombination fraction values with a decrease in LOD score indicates no genotyping problem in the markers.

**Supplementary Figure 4.** Graphical representation of genotypes of the 21 chromosomes. Red, alleles from ‘Norin 61’; blue, alleles from synthetics derived from KU-2039; white, missing information. Black vertical lines indicate boundaries between chromosomes. Recombination blocks (chromosomes) are clearly defined.

**Supplementary Figure 5.** Heat map of pairwise recombination fractions (above the diagonal) and LOD scores (below the diagonal) for all marker combinations. Low recombination fraction values and high LOD scores are shown in yellow while blue color represents converse.

**Supplementary Figure 6.** QTL mapping for days to heading using the ICIM-ADD method in the BIL population. The significant LOD on chromosome 2D agrees with the well-known QTL for days to heading (*Ppd-D1* gene).

**Supplementary Figure 7.** Mapping of QTLs for germination percentage G% in season one S1 and season two S2 with the ICIM-ADD method. (A) The identified QTL for G%_S1; (B) the specific position of the QTL for G%_S1 on chromosome 5D; (C) the identified QTL for G%_S2; (D) the specific position of the QTL for G%_S2 on chromosome 1D; (E) the specific position of the QTLs for G%_S2 on chromosome 5D. The dash-dotted blue lines indicate the threshold of the LOD score at 3.0.

**Supplementary Figure 8.** Mapping of QTLs for germination index GI in season two S2 with the ICIM-ADD method. (A) The identified QTL for GI_S2; (B) the specific position of the QTL for GI_S2 on chromosome 5B; (C) the specific position of the QTL for GI_S2 on chromosome 5D. The dash-dotted blue lines indicate the threshold of the LOD score at 3.0.

**Supplementary Figure 9.** Genome-wide association analysis of seed dormancy. Manhattan plots of (A) germination percentage season one (G%_S1), (B) germination percentage season two (G%_S2), and (C) the combined germination percentage in S1 and S2 (G%) in the BIL population. The green solid line indicates the significance of the Bonferroni correction threshold, and the green dashed line indicates the false discovery rate. Individual chromosomes are indicated by different colors.

**Supplementary Figure 10.** Genome-wide association analysis of seed dormancy. Manhattan plots of (A) germination index season one (GI_S1), (B) germination index season two (GI_S2), and (C) the combined germination index (GI) in the BIL population. The green solid line indicates the significance of the Bonferroni correction threshold, and the green dashed line indicates the false discovery rate. Individual chromosomes are indicated by different colors.

**Supplementary Figure 11.** Distribution of 174 markers on chromosome 5D. Red, markers flanking *QSd.alrc-5D*, the dormancy QTL identified in this study. *Qdor.hzau-5D*, the previously reported dormancy QTL.

**Supplementary Figure 12.** The temperature differences between seasons 2021 and 2022 at the grain development stage (April, May, and June).

**Supplementary Table 1.** Origin of *Aegilops tauschii* accessions that contributed dormancy genes and the pedigrees of their dormant descendants identified in the population of multiple synthetic derivatives

**Supplementary Table 2.** Germination percentage (G%) and germination index (GI) of the 166 lines in each of the two seasons (S1 and S2) and in both seasons combined.

**Supplementary Table 3.** Marker alignment in the sub-genomes A, B, and D with their genetic and physical positions on each chromosome.

**Supplementary Table 4.** Genes found between the QTL flanking markers (AMP0017090–AMP0004316).
