# Supplementary figures and images for: Mining Aegilops tauschii genetic diversity in the background of bread wheat revealed a novel QTL for seed dormancy
Source: Front Plant Sci. 2023 Nov 30;14:1270925. doi: 10.3389/fpls.2023.1270925 (PMC10723804; doi:10.3389/fpls.2023.1270925)

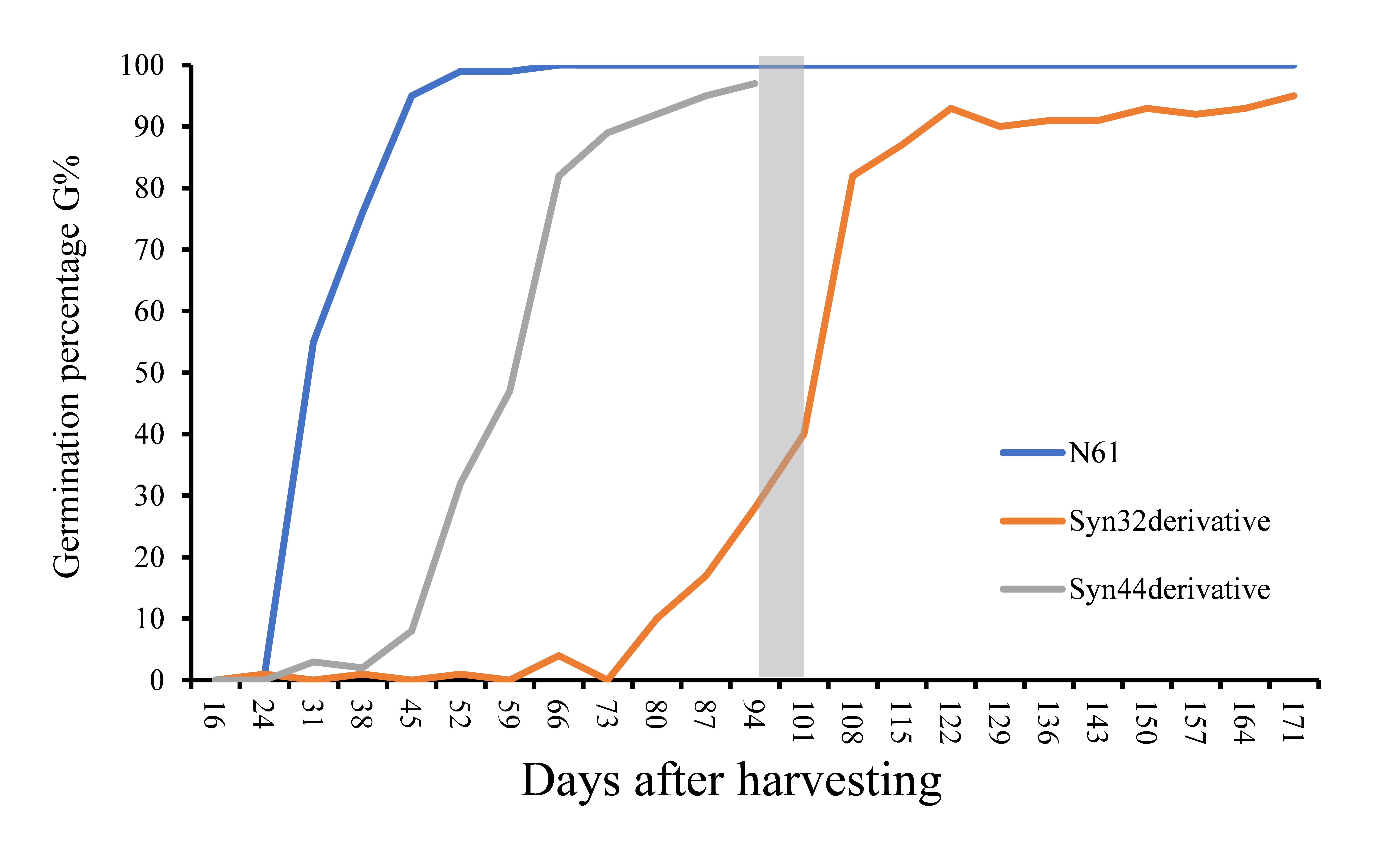

Supplement: Supplementary file 2 [file Image_1.jpeg]

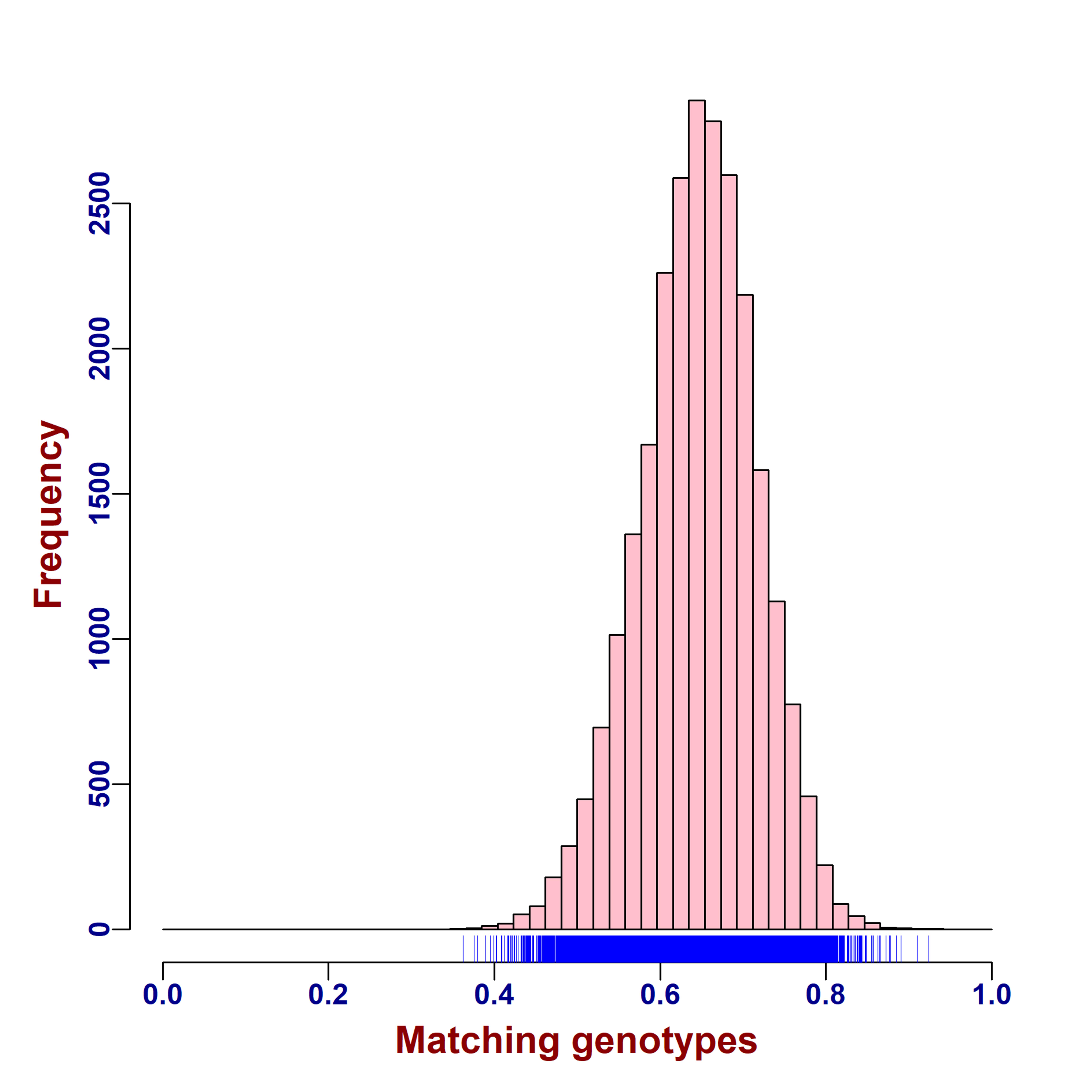

Supplement: Supplementary file 3 [file Image_2.jpeg]

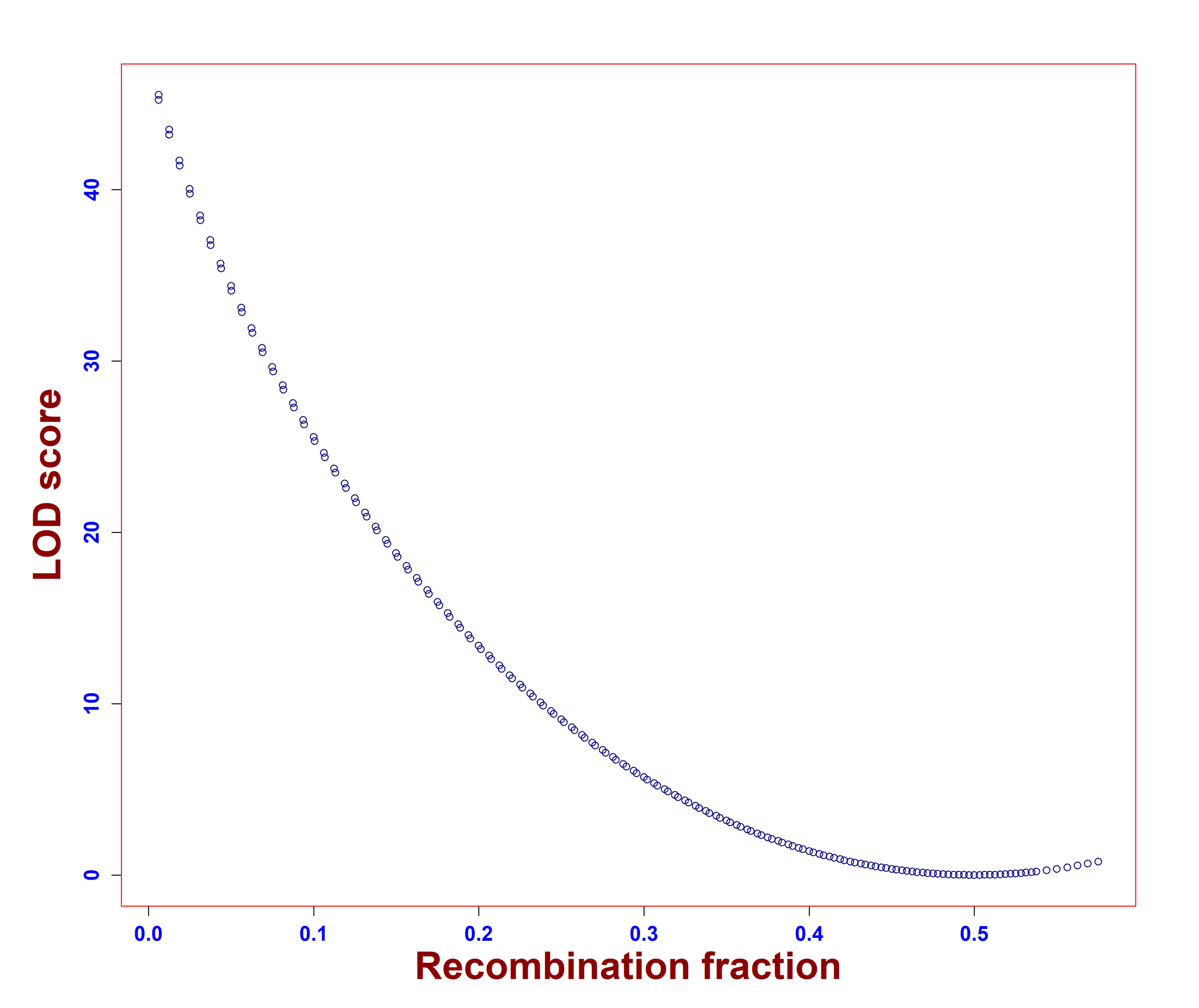

Supplement: Supplementary file 4 [file Image_3.jpeg]

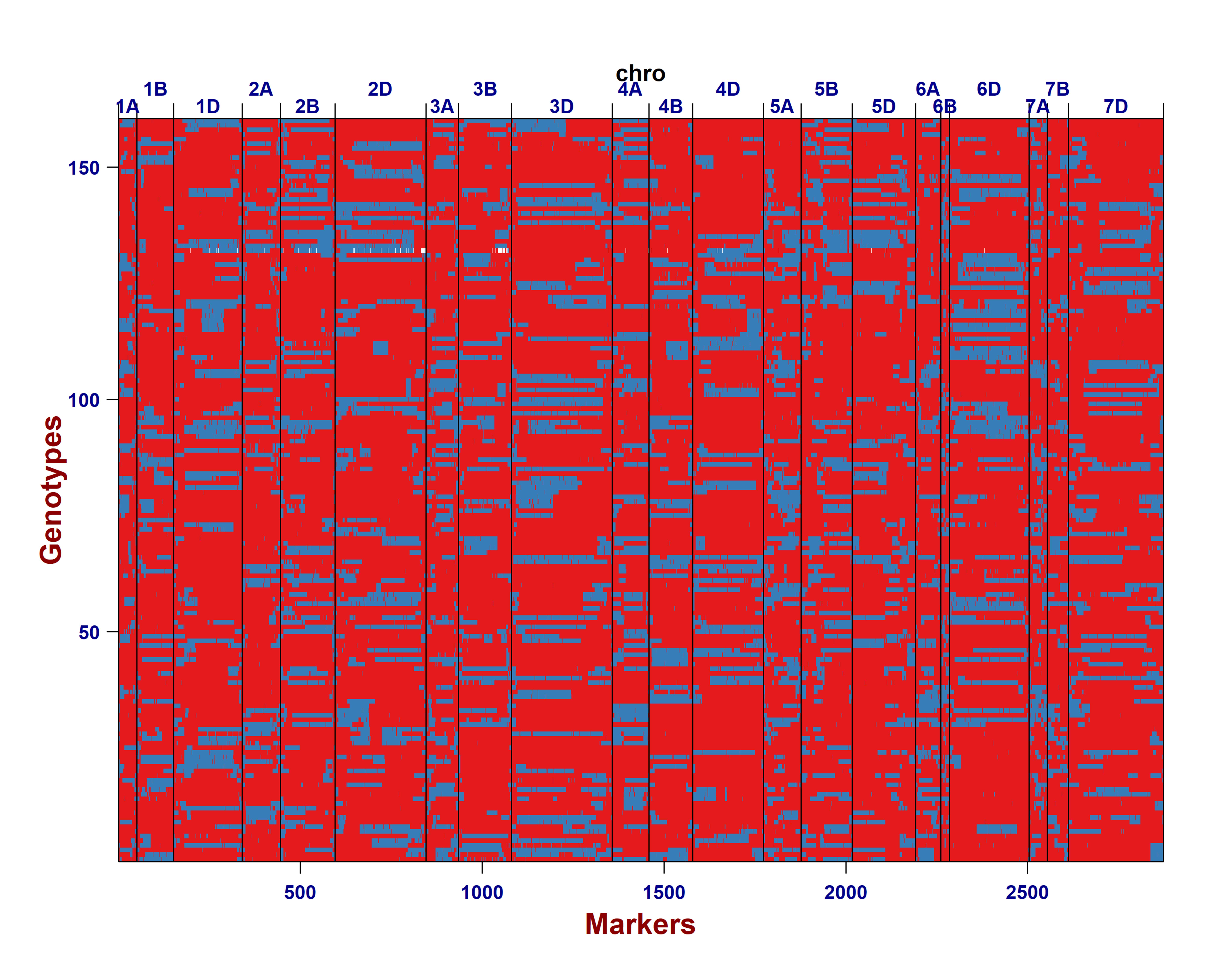

Supplement: Supplementary file 5 [file Image_4.jpeg]

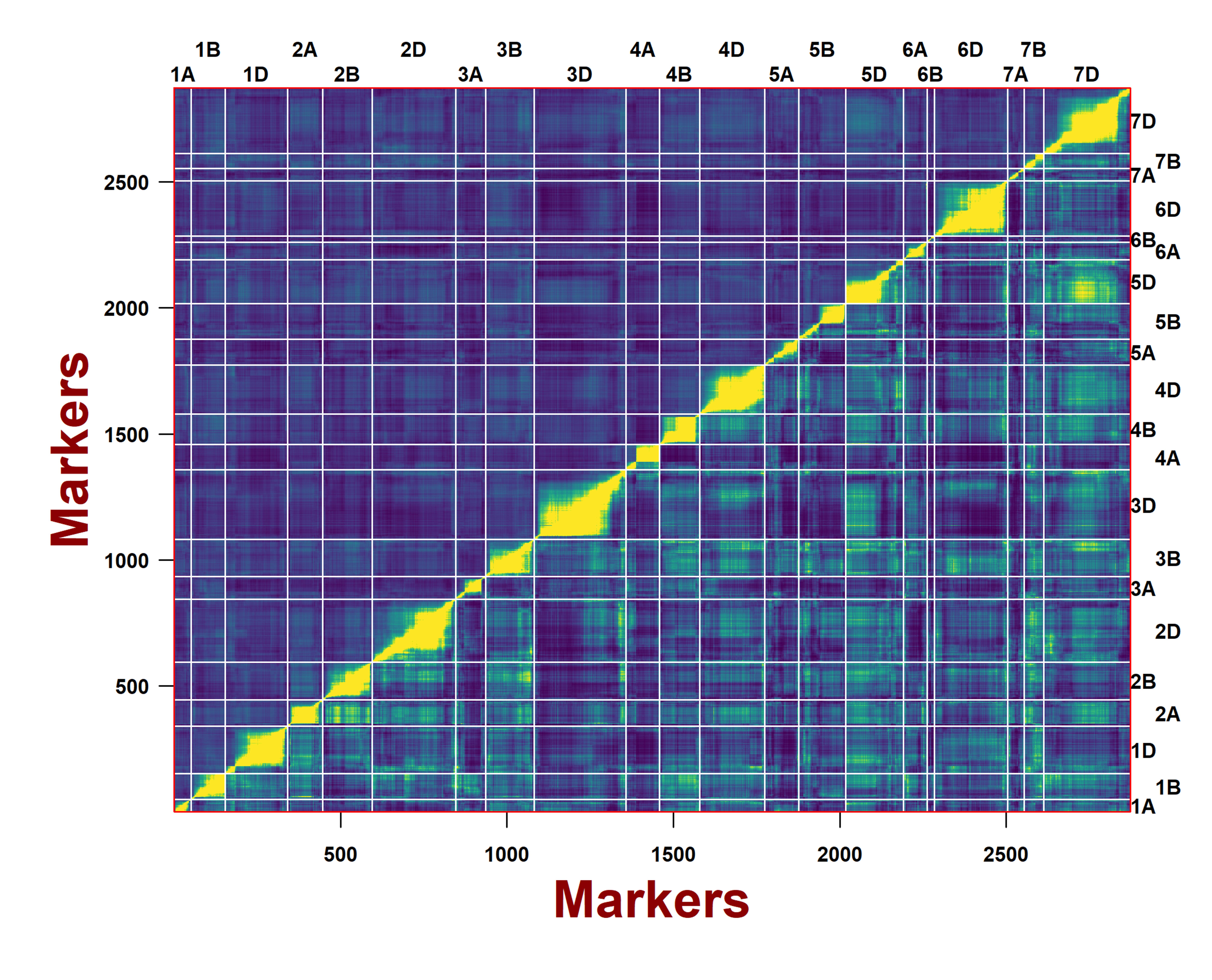

Supplement: Supplementary file 6 [file Image_5.jpeg]

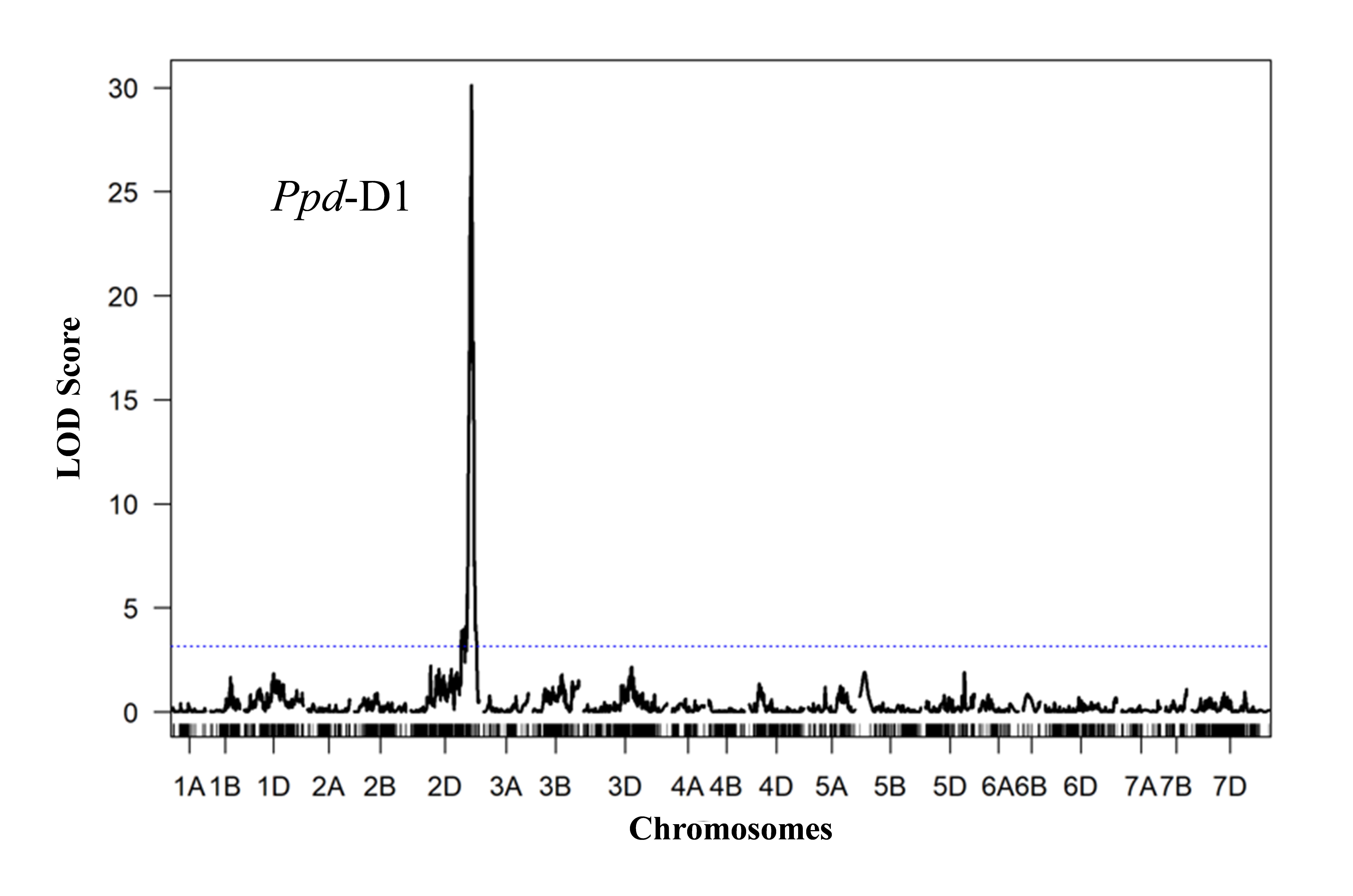

Supplement: Supplementary file 7 [file Image_6.jpeg]

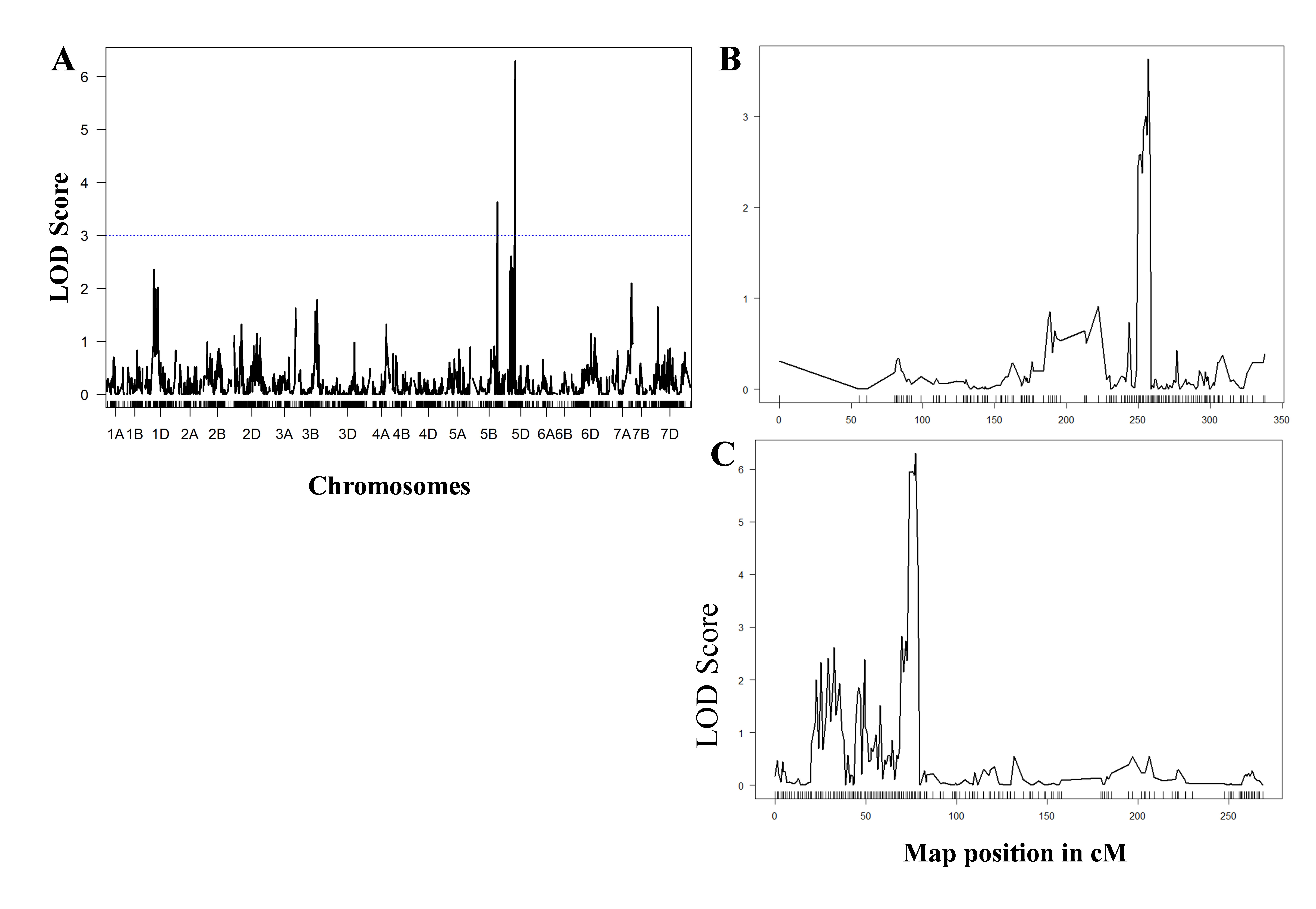

Supplement: Supplementary file 9 [file Image_8.jpeg]

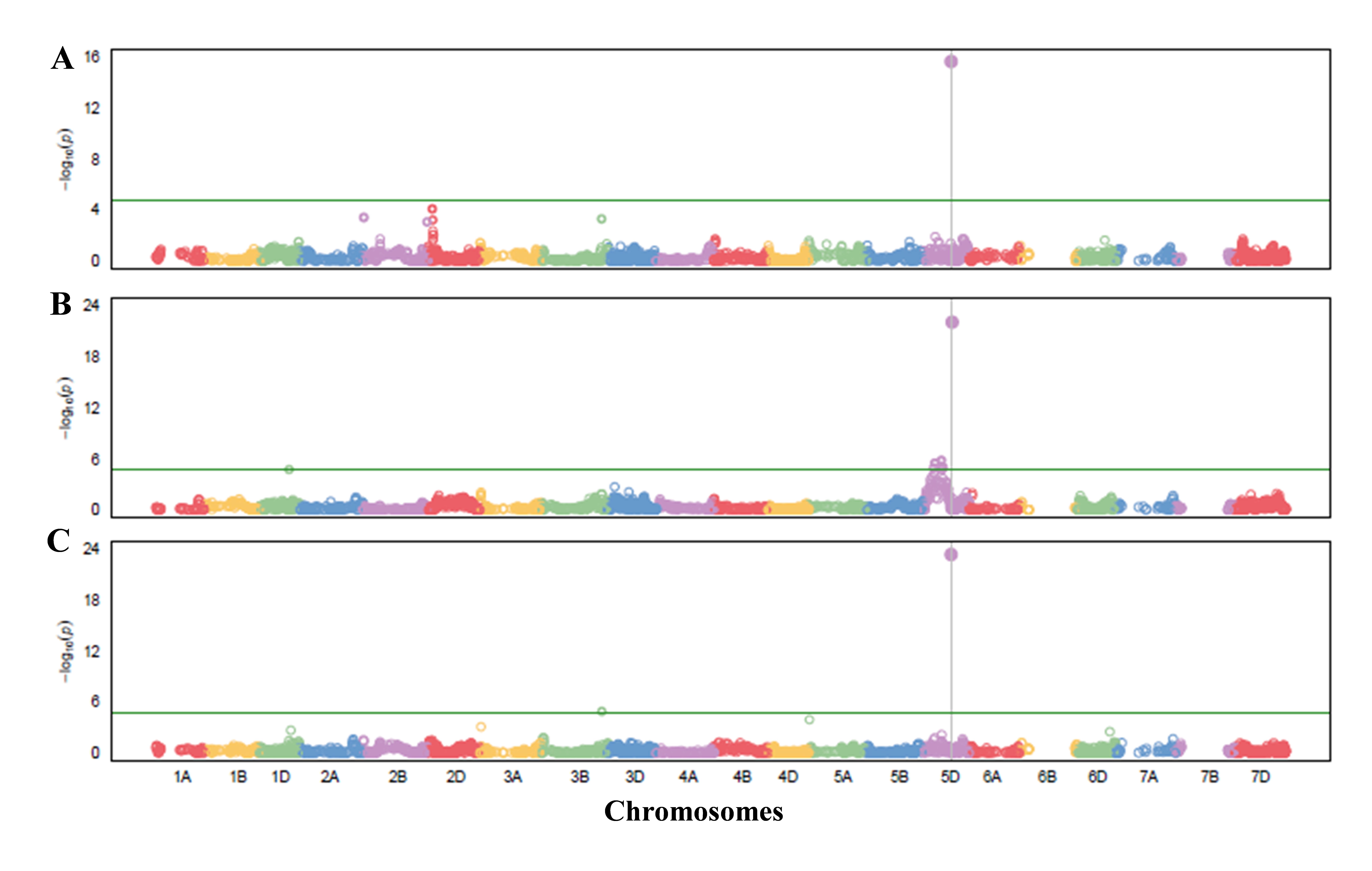

Supplement: Supplementary file 10 [file Image_9.jpeg]

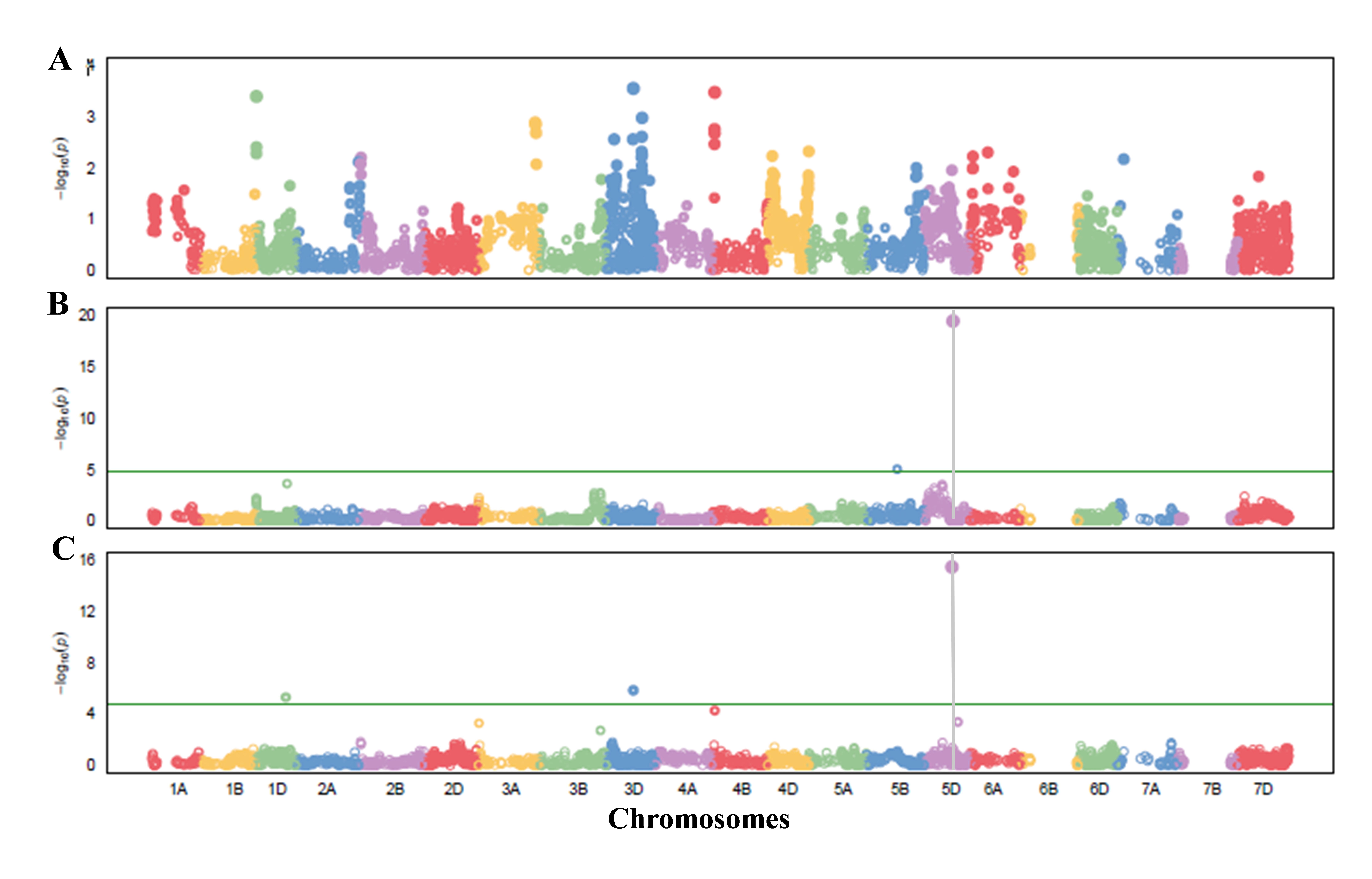

Supplement: Supplementary file 11 [file Image_10.jpeg]

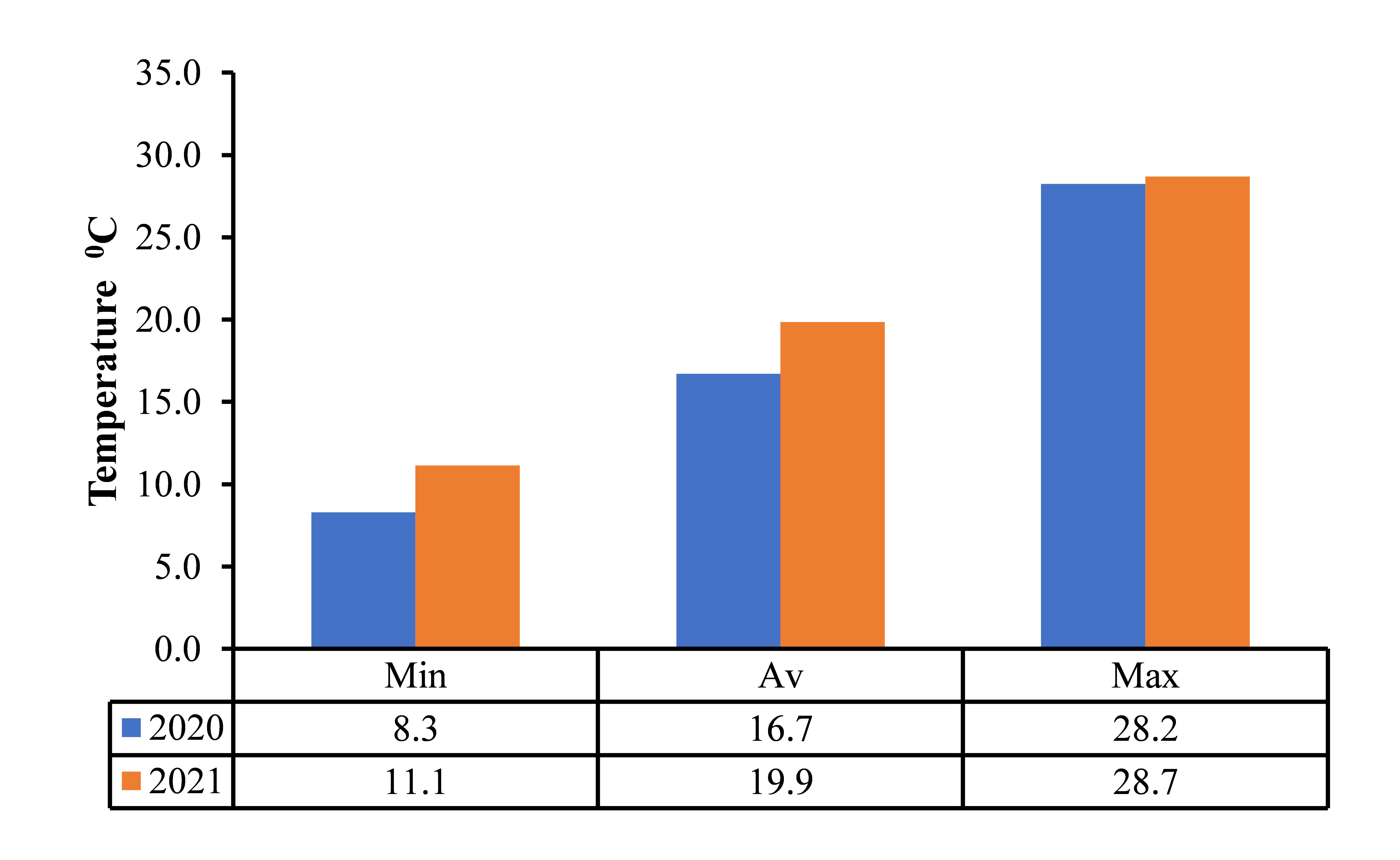

Supplement: Supplementary file 13 [file Image_12.jpeg]
